# Dietary licorice improves carcass yield, hepatocellular protection, gut health, oxidative status, and cecal bacterial balance in broiler chickens

**DOI:** 10.1016/j.vas.2026.100892

**Published:** 2026-10-06

**Authors:** Chidozie F. Egbu, Christiaan J. Smit

**Affiliations:** a Department of Agriculture and Animal Health, College of Agriculture and Environmental Sciences, University of South Africa, Florida Park, Roodepoort, 1709, South Africa; b Department of Agricultural Science, Faculty of Vocational and Technology Education, Alvan Ikoku Federal University of Education, Owerri, P.M.B. 1033, Nigeria

**Keywords:** Antibiotic alternative, Gut microbiota, Immunology, Meta-analysis, Phytotherapy, Poultry nutrition

## Abstract

Over the past decade, phytotherapy such as licorice has attracted research as an alternative to antibiotic growth promoters in broiler production, driven by rising concerns over antimicrobial resistance. However, the consistency of its effects across productive, physiological, and health outcomes remains inconsistent. This meta-analysis quantitatively synthesized published experimental evidence on the effects of dietary licorice on growth performance, carcass yield, blood indices, visceral organs, oxidative status, intestinal morphology, and cecal bacterial balance in broiler chickens. A systematic literature search identified studies published between 2010 and 2025, and effect sizes were computed (Hedges' *g*; 95% confidence interval). Pooled estimates were derived under the random-effects model using the restricted maximum likelihood estimator, with between-study heterogeneity characterized by *I² and* the *Q*-statistic. Univariable mixed-effects meta-regression was conducted to identify study-level moderators of treatment response, and publication bias was assessed using a funnel plot and the Rosenberg fail-safe N criterion. Dietary licorice did not affect feed intake or the feed conversion ratio, but it did increase body weight gain (BWG; SMD = 0.289; *P* = 0.002), breast, thigh, gizzard, and bursa weights (*P* < 0.05). Gut integrity also improved with the villus height/crypt depth ratio (SMD = 0.931; *P* = 0.041), and *Lactobacillus* spp. counts (SMD = 0.266; *P* = 0.003) increased, while *Escherichia coli* populations were suppressed (SMD = −1.737; *P* < 0.001). Overall, the findings support that licorice enhances body weight gain, with route, duration, sex, and strain moderating efficacy.

## Highlights

• Dietary licorice improved body weight gain and carcass yield in broilers.
• Licorice supplementation reduced cholesterol, glucose, and malondialdehyde levels.
• Licorice lowers *Escherichia coli* and enhances the villus height/crypt depth ratio.

## Introduction

The extensive use of antibiotic growth promoters (AGPs) to enhance poultry performance has been restricted by many countries (Salim et al., 2018) due to concerns related to antimicrobial resistance and food safety (Etienne et al., 2025). These total or partial bans have prompted the search for sustainable alternatives. Against this backdrop, bioactive compounds derived from plants have attracted considerable research attention, owing to their documented growth-promoting, antimicrobial, antioxidant, and digestive-stimulating properties (Taghizadeh et al., 2024). Among these plants, licorice (*Glycyrrhiza glabra* L.), a perennial legume of the Fabaceae family cultivated for its sweet roots, is native to the Mediterranean basin and central to southwestern Asia (Karkanis et al., 2016). Licorice has been documented to contain liquiritin, glycyrrhizin, glabridin, licoflavone, licochalcone, and saponins, which have antibacterial, antioxidant, anti-inflammatory, and immunomodulatory properties (Babich et al., 2022; Sohail et al., 2018). Mechanistically, licochalcone has been shown to activate Nrf2, which supports its antioxidant mode of action (Lv et al., 2019). Glycyrrhizin attenuates pro-inflammatory responses by interfering with receptor signaling (Schröfelbauer et al., 2009). Qu et al. (2025) noted that glabridin restores the sensitivity of colistin against *mcr*-*1*-positive *E. coli* through the polypharmacology mechanism. These results provide a plausible biological basis for the growth and gut health effects evaluated in this meta-analysis.

Building on this pharmacological profile, dietary 0.2 g licorice essential oil/kg increased feed efficiency while reducing *E. coli* in broiler chickens after 42 days (Geravand et al., 2021). Similarly, licorice powder supplemented at 7.5 g/kg in the diets of Cobb 500 chickens enhanced body weight gain (BWG) and feed conversion ratio (FCR) while decreasing serum cholesterol, triglyceride, and low-density lipoprotein (Kairalla & Alshelmani, 2025). Notwithstanding, Beski et al. (2019) reported no variation in the feed intake (FI), FCR, dressing percentage, carcass cuts, visceral organs, and intestinal morphology of Ross 308 chickens administered 0.9 g/kg aqueous licorice extract for 35 days. Similarly, in Ross 308 broilers, inclusion of licorice oil at 3 g/kg of the diet did not influence growth performance, immune organs, or carcass indices but reduced serum cholesterol and glucose (Dheyauldeen et al., 2023). These discrepancies likely reflect the substantial heterogeneity of experimental conditions across primary studies, including variation in licorice form, supplementation route, inclusion level, bird strain, and duration of inclusion. More recently, Kairalla & Alshelmani (2025) reported the growth performance and serum biochemistry benefits of dietary licorice powder supplemented at 2.5–7.5 g/kg in Cobb 500 broilers. Nonetheless, the variability in response magnitude across inclusion levels has reinforced the need for a quantitative synthesis of the available evidence base.

Despite the accumulating primary studies, no meta-analysis has quantitatively synthesized the effects of licorice supplementation on growth performance, carcass yield, blood indices, visceral and immune organs, intestinal morphology, oxidative status, and cecal bacterial balance within a single unified analytical framework. This gap is consequential, as without a pooled quantitative estimate that accounts for between-study heterogeneity, researchers cannot reliably translate the experimental evidence into actionable supplementation guidance for the poultry industry. Meta-analysis aggregates effect sizes across independent studies, quantifying heterogeneity, and enabling systematic exploration of sources of variation through subgroup analysis and meta-regression (Morris & DeShon, 2002). Therefore, this study aimed to quantify the effects of dietary licorice on broiler growth performance, carcass yield, blood indices, organ indices, intestinal morphology, oxidative status, and cecal bacterial balance using a meta-analysis. Furthermore, determine whether inclusion level, route, duration, form, sex, and strain explain variation in dietary licorice efficacy. We hypothesized that dietary licorice would improve growth performance, carcass yield, intestinal morphology, and beneficial cecal bacterial populations while reducing oxidative stress markers and pathogenic bacterial load in broiler chickens. The magnitude of these responses would be moderated by inclusion level, form, route, duration, sex, and strain. Thereby, provide scientific evidence for optimizing licorice supplementation in broiler production as an alternative to AGP.

## Materials and methods

### Studies inclusion and exclusion strategy

A systematic literature search of studies between 01/01/2010 and 31/12/2025 was done following the PRISMA protocols, with searches conducted from 1 April 2026 to 30 April 2026. AGORA, Google Scholar, PubMed, Scopus, and Web of Science were the databases the search was performed across. The search string used across all databases was licorice OR liquorice OR “Glycyrrhiza glabra*” AND “broiler chicken” AND “growth performance” OR “body weight gain” OR “feed intake” OR “feed efficiency” OR “breast yield” OR “thigh yield” OR liver OR gizzard OR spleen OR thymus OR “bursa of Fabricius” OR “red blood cell” OR “white blood cell” OR “hemoglobin” OR “total protein” OR albumin OR globulin OR cholesterol OR glucose OR “aspartate aminotransferase” OR “alanine aminotransferase” OR catalase OR glutathione OR malondialdehyde OR “superoxide dismutase” OR “crypt depth” OR “villus height” OR “villus width” OR “villus height/crypt depth ratio” OR “*E. coli*” OR “*Lactobacillus*”. Duplicate records identified across databases were removed before screening using Rayyan’s systematic review software. Studies were included in the meta-analysis following the PICO framework. Only broiler chickens were the population of interest. The intervention must be licorice supplementation in any form. The comparison involved a control group reared on a basal diet without licorice supplementation or an AGP. Challenge studies (Eimeria challenge, mycotoxin studies, and heat stress) were excluded from this study. The database search yielded 2607 records, of which 812 duplicates were removed. The remaining 1795 records were screened for title/abstract, with 1641 excluded at this stage. Retrieval was sought for 154 reports, of which 13 could not be retrieved due to open access restrictions and no response from authors after requests. The remaining 141 full-text articles were assessed for eligibility. Thereafter, 120 articles were excluded for the following reasons: not broiler chickens (n = 28), licorice not used or not the main intervention (n = 30), no appropriate control group (n = 22), challenge studies (n = 31), and outcomes of interest not reported (n = 9). The qualitative synthesis and meta-analysis included 21 studies (Fig. 1). The outcomes evaluated were growth performance [FI (g), BWG (g), or FCR], carcass yield [breast (g) or thigh (g)], visceral organ weights [liver (g), gizzard (g), spleen (g), thymus (g), or bursa of Fabricius (g)], hematology [red blood cell (RBC; ×10¹²/L), white blood cell (WBC; ×10⁹/L), or hemoglobin (g/dL)], serum biochemistry [total protein (TP; g/L), albumin (g/L), globulin (g/L), cholesterol (mmol/L), or glucose (mmol/L)], liver enzymes [aspartate aminotransferase (AST; U/L) or alanine aminotransferase (ALT; U/L)], oxidative status [catalase (U/mL), glutathione (GSH; mmol/L), malondialdehyde (MDA; nmol/mL), or superoxide dismutase (SOD; U/mL)], intestinal morphology [crypt depth (CD; µm), villus height (VH; µm), villus width (VW; µm), or VH/CD)], and cecal bacterial count [*E. coli* (Log cfu/g) or *Lactobacillus spp*. (Log cfu/g)].

**Fig. 1:**
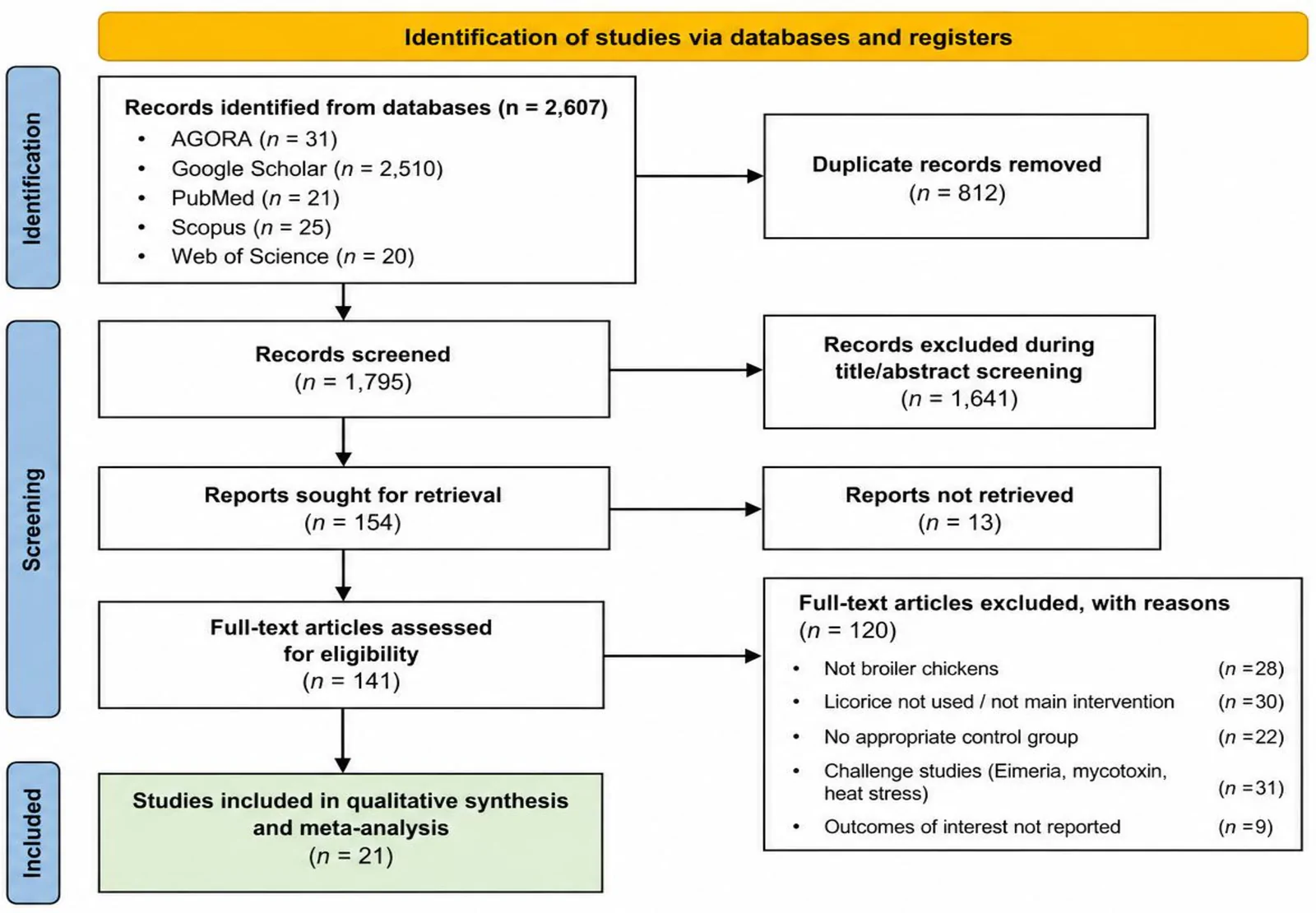
PRISMA flow diagram.

### Risk of bias assessment

The methodological quality of the 21 included studies was independently assessed by two reviewers using SYRCLE's Risk of Bias tool for animal intervention studies (Hooijmans et al., 2014). Each study was evaluated across the sequence generation, baseline characteristics, allocation concealment, random housing, blinding of researchers, random outcome assessment, blinding of outcome assessors, incomplete outcome data, selective outcome reporting, and other sources of bias. Each domain in each study was rated as low, unclear, or high risk based on whether the relevant procedure was described. Studies were expected to specify whether the measures described were adequate to prevent bias. Disagreements between reviewers were resolved by consensus. No study was excluded from the quantitative synthesis based on its risk-of-bias profile. Instead, the assessment was used to contextualize confidence in the pooled estimates and to inform the interpretation of heterogeneity.

### Data extraction

Two reviewers independently screened articles for eligibility, with discrepancies resolved by consensus. Information on the surname of the first author, year of publication, country, continents, number of birds, number of treatments, strain, sex, inclusion level, inclusion route, inclusion form, inclusion duration, and outcomes were extracted from the 21 articles that met the inclusion criteria (Table 1). For the Excel sheet used for analysis, data on the control group mean, control standard deviation, control sample size, experimental group mean, experimental standard deviation, and experimental sample size were also included. Where standard error (SE) was reported in a trial study in place of standard deviation (SD), the SE values were converted into SD using SD=SE×√n where *n* is the number of replicates per each treatment group.

**Table 1:** Overview of publications that met the inclusion criteria.

No	Authors	Country	Continent	NB	NT	Strain	Sex	IL (g/kg)	Form	ID (d)	IR	Outcomes
1	Alhujaili et al. (2025)	Egypt	Africa	210	3	Cobb	Unsexed	0–2	Extract	28	DW	1, 2, 3, 6, 7, 8, 14, 15, 16, 17, 20, 21, 23, 24, 29, 30
2	Kairalla & Alshelmani (2025)	Libya	Africa	192	4	Cobb	Unsexed	0–7.5	Powder	42	Feed	1, 2, 3, 11, 12, 13, 14, 15, 16, 17, 18, 19, 23, 24
3	Rashad et al. (2025)	Egypt	Africa	280	4	Cobb	Unsexed	0–3	Extract	30	DW	1, 2, 3, 6, 7, 8, 14, 15, 16, 17, 20, 21, 23, 24, 29
4	Tang et al. (2025)	China	Asia	540	5	Cobb	Unsexed	0–4	Extract	42	Feed	1, 2, 3, 14, 15, 16, 17, 19, 20, 21, 22, 23, 24, 25, 26, 28
5	Gowthaman et al. (2024)	India	Asia	525	5	Kadaknath	Unsexed	0–7	Powder	105	Feed	1, 2, 3, 6, 7, 14, 15, 16, 17, 23, 24, 25, 26, 27, 28, 29, 30
6	Li et al. (2024)	China	Asia	210	2	Liangfenghua	Male	0–1	Extract	84	Feed	1, 2, 3, 19, 20, 21
7	Shaban et al. (2024)	Iran	Asia	120	3	Ross	Unsexed	0–0.2	Powder	42	Feed	1, 2, 3, 4, 6, 8, 9, 14, 15, 16, 17, 18, 25, 26, 27, 28, 29, 30
8	Dheyauldeen et al. (2023)	Iraq	Asia	160	4	Ross	Unsexed	0–3	Oil	42	Feed	1, 2, 3, 4, 6, 8, 9, 17, 18
9	Toson et al. (2023)	Egypt	Africa	320	4	Ross	Unsexed	0–3	Extract	35	Feed	1, 2, 3, 6, 7, 11, 12, 13, 14, 15, 16, 17, 19, 20, 21, 22, 23, 24
10	Ullah et al. (2023)	Pakistan	Asia	120	4	Unknown	Unsexed	0–2	Extract	35	DW	1, 2, 3, 25, 26, 27, 28
11	Abo-Samaha et al. (2022)	Egypt	Africa	180	3	Cobb	Unsexed	0–0.8	Extract	35	DW	1, 2, 3, 6, 7, 8, 9, 10, 14, 15, 16, 17, 18, 19, 20, 22, 23, 24, 25, 26, 27, 28
12	Geravand et al. (2021)	Iran	Asia	375	3	Unknown	Unsexed	0–0.4	Oil	42	Feed	1, 2, 3, 11, 12, 14, 15, 16, 17, 29, 30
13	Noor et al. (2021)	Iraq	Asia	180	4	Ross	Unsexed	0–15	Extract	42	DW	1, 2, 3, 4, 5, 6, 7
14	Ibrahim et al. (2020)	Egypt	Africa	500	5	Ross	Male	0–2	Extract	35	Feed	1, 2, 3, 6, 7
15	Iqbal et al. (2020)	Pakistan	Asia	150	5	Cobb	Unsexed	0–25	Extract	35	Feed	1, 2, 3, 4, 5, 6, 7, 13, 17
16	Beski et al. (2019)	Iraq	Asia	160	4	Ross	Unsexed	0–0.9	Extract	35	DW	1, 2, 3, 4, 5, 6, 8, 9, 25, 26, 28
17	Rashidi et al. (2019)	Iran	Asia	144	2	Ross	Unsexed	0–0.5	Extract	42	Feed	1, 2, 3, 4, 5, 6, 7, 17, 18,
18	Naser et al. (2017)	Iran	Asia	400	4	Cobb	Unsexed	0–0.3	Extract	42	DW	1, 2, 3, 4, 5, 6, 17, 18
19	Jagadeeswaran & Selvasubramanian (2014)	India	Asia	36	2	Cobb	Unsexed	0–1	Extract	42	Feed	12, 14, 15, 16
20	Naser et al. (2013)	Iran	Asia	320	4	Cobb	Unsexed	0–0.3	Extract	42	DW	1, 2, 3, 6, 8, 9, 10,
21	Sedghi et al. (2010)	Iran	Asia	480	4	Ross	Male	0–2	Extract	49	Feed	3, 4, 6, 8, 11, 12, 17, 18

NB = number of birds; NT = number of treatments; IL = inclusion level; ID = inclusion duration; DW = drinking water; 1 = feed intake; 2 = body weight gain; 3 = feed conversion ratio; 4 = breast; 5 = thigh; 6 = liver; 7 = gizzard; 8 = spleen; 9 = bursa of Fabricius; 10 = thymus; 11 = red blood cell; 12 = white blood cell; 13 = hemoglobin; 14 = total protein; 15 = albumin; 16 = globulin; 17 = cholesterol; 18 = glucose; 19 = glutathione; 20 = malondialdehyde; 21 = superoxide dismutase; 22 = catalase; 23 = aspartate aminotransferase; 24 = alanine aminotransferase; 25 = crypt depth; 26 = villus height; 27 = villus width; 28 = villus height/crypt depth ratio; 29 = *Escherichia coli*; 30 = *Lactobacillus* species

Most contributing studies expressed inclusion levels in g/kg of diet. Where studies reported inclusion levels as a percentage of diet, values were converted to g/kg by multiplying by 10 (e.g., 0.1% = 1 g/kg). Where supplementation levels were expressed in mg/kg, values were converted to g/kg by dividing by 1000 (e.g., 500 mg/kg = 0.5 g/kg). The only two studies on oil reported inclusion levels in percentages and mg/kg. When studies reported multiple levels of licorice inclusion against a common control group, each treatment comparison was included as a separate effect size, with the shared control group divided proportionally across comparisons to avoid the unit-of-analysis error associated with the control being counted twice in the pooling model following Cochrane’s recommendation (Garritty et al., 2024). Each level-control comparison was treated as an independent comparison (*n*^c^), while the study itself was counted once as a unique study (*n*^s^).

### Data analysis

The OpenMEE and *MetaWin*softwares were used to analyze all the data. Pooled results were estimated as a standardized mean difference (SMD; Hedges’ *g*) with a 95% confidence interval (CI) using the random-effects model. For all outcomes, the SMD was computed as the difference between the licorice group and the control group (treatment minus control). Thus, a positive SMD reflects a licorice-induced increase, and a negative SMD reflects a licorice-induced decrease relative to control. A random-effects model with restricted maximum likelihood estimation was applied to all meta-analyses. The statistical model was specified as:

$$Y_{\text{ij}}=\mu +T_{i}+u_{i}+e_{\text{ij}}$$

where *Y*_ij_ is the observed effect size (SMD) for the *j*^th^ comparison within the *i*^th^ study; μ is the overall grand mean (pooled effect); *T*i is the fixed effect of licorice treatment relative to a control without licorice; *u*_i_ ∼ N(0, τ²) is the random effect capturing true between-study heterogeneity in effects (τ²); and *e*_ij_ ∼ N(0, σ²) is the residual within-study sampling error.

The Cochran *Q* test and its associated *p*-value and heterogeneity (*I²*) were used to characterize between-study heterogeneity. Consistent with Cochrane Handbook guidance on investigating *I²* (Deeks et al., 2023), subgroup, meta-regression, and publication-bias analyses were prespecified to be conducted only for outcomes with *P <* 0.05 and *I*² > 50%. To guard against overfitted moderator estimates, this step was further restricted to outcomes with a minimum of 10 studies (Higgins et al., 2019). Subgroup and meta-regression analysis was conducted based on the following covariates of interest evaluated as potential moderators: inclusion level (g/kg), inclusion route (drinking water or feed), inclusion duration (days), inclusion form (powder, extract, or oil), broiler sex (male or unsexed), and broiler strain. Moderators were evaluated one at a time using univariable mixed-effects meta-regression. The subgroup model extended the base random-effects specification as follows:

$$Y_{i}=\mu +\beta_{1}X_{1i}+u_{i}+e_{i}$$

Where *Y*_i_ is the effect size for study *i; β*_1_*X*_1i_ are the regression coefficients for each moderator variable (inclusion level, inclusion route, inclusion duration, inclusion form, sex, and strain); and *u*_i_ and *e*_i_ retain their definitions as above. The meta-regression used the R² analog to observe the extent to which covariates in the model have explained the unexplained variance in true effects. Publication bias was evaluated using funnel plots, Egger’s regression test, the trim-and-fill method, and Rosenberg’s weighted fail-safe N (*N_fs_*). To assess the robustness of the meta-analytic findings, the calculated *N_fs_* was compared against the standard threshold of *5N_study_* + 10 (where *N_study_* is the number of included studies). The meta-analysis results were adjudged robust against publication bias if the calculated *N_fs_* exceeded this threshold. Sensitivity of the pooled estimates was evaluated using leave-one-out analysis, in which each study was systematically excluded, and the remaining studies were re-pooled to assess whether any single study exerted a disproportionate influence on the pooled results. Statistical significance for all tests was set at *P* < 0.05.

## Results

### Description of the studies in the meta-analysis

A total of 21 articles (between 2010 and 2025) met the selection criteria for the present meta-analysis, including 5602 birds across seven countries within Africa and Asia (Fig. 2; Table 1). Geographically, Iran (*n* = 6) contributed the most studies, followed by Egypt (*n* = 5), Iraq (*n* = 3), China (*n* = 2), India (*n* = 2), Pakistan (*n* = 2), and one study from Libya. The number of birds and treatments per study ranged from 36 to 540 and 2 to 5, respectively. Cobb (*n* = 9) was the most investigated of the 21 studies. The forms of licorice inclusion across studies were powder (*n* = 3), oil (*n* = 2), and extract (*n* = 16), with levels ranging from 0.1 to 25 g/kg. Growth performance (*n* = 19) outcomes were the most reported endpoints.

**Fig. 2:**
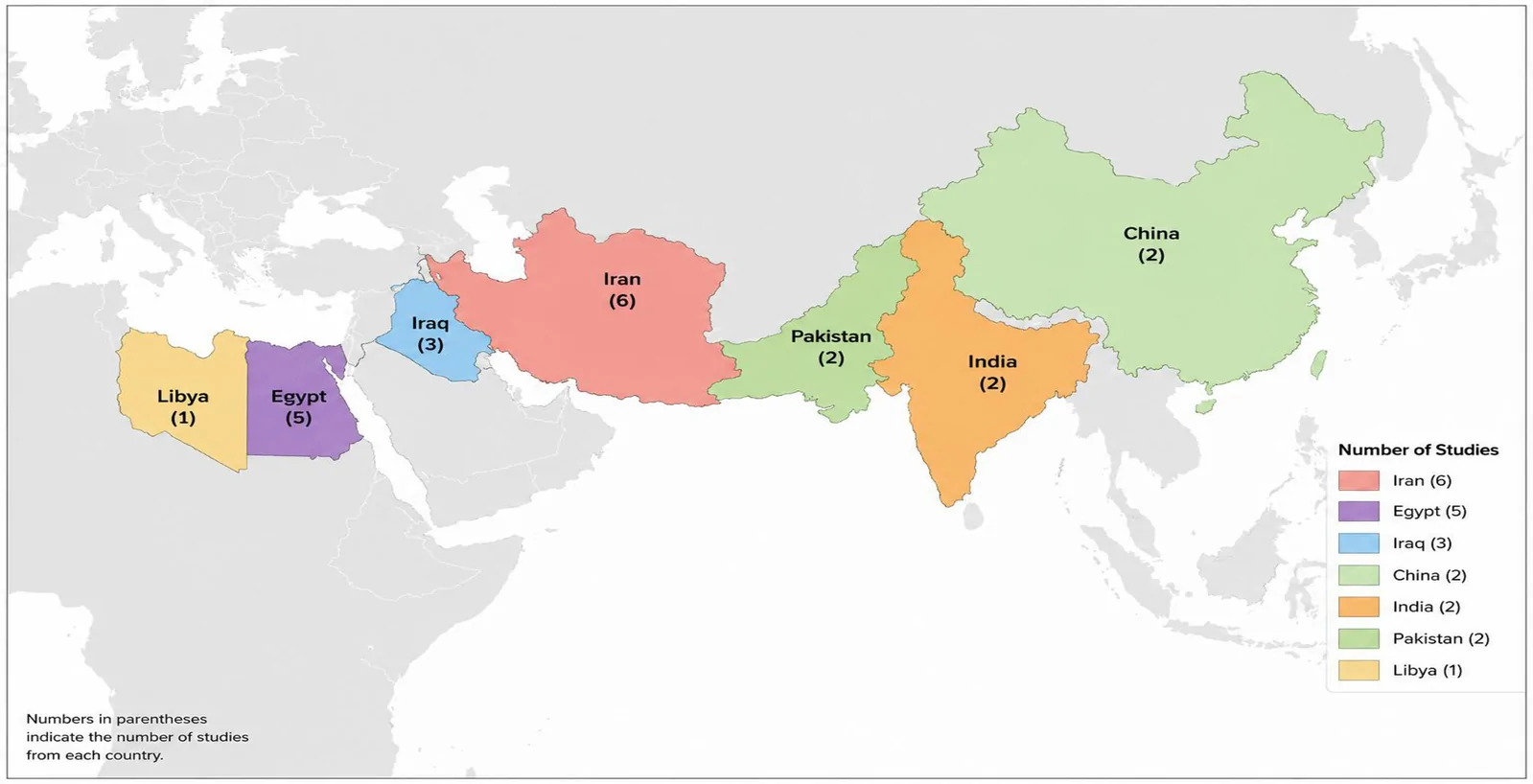
Contributing studies distribution map.

### Risk of bias outcome

The per-study risk-of-bias matrix is presented in Fig. 3, while the aggregate distribution across domains is described in Fig. 4. Sequence generation, allocation concealment, random housing, and blinding of both caregivers/researchers and outcome assessors were consistently unreported at a level of detail sufficient for judgment. Instead, studies stated that animals were "randomly allocated" without describing the randomization mechanism. Likewise, none reported concealment of allocation, randomized pen/cage placement, or blinding at any stage. Selective outcome reporting was also rated unclear across all studies because none had a registered or pre-specified protocol to verify reported outcomes. Baseline comparability and other sources of bias were rated low risk throughout. Incomplete outcome data was rated low risk in 19 studies, which reported mortality/attrition. Nevertheless, two studies did not report attrition and were rated unclear. Random outcome assessment was the only domain showing a mixed pattern. Studies that randomly selected animals for terminal sampling had 13 and were rated low risk. Four studies used a non-random selection method to choose animals that were “close to” the group average weight for carcass or organ measurements, and these studies were rated as high risk. The remaining studies did not describe a selection method and were rated as unclear.

**Fig. 3:**
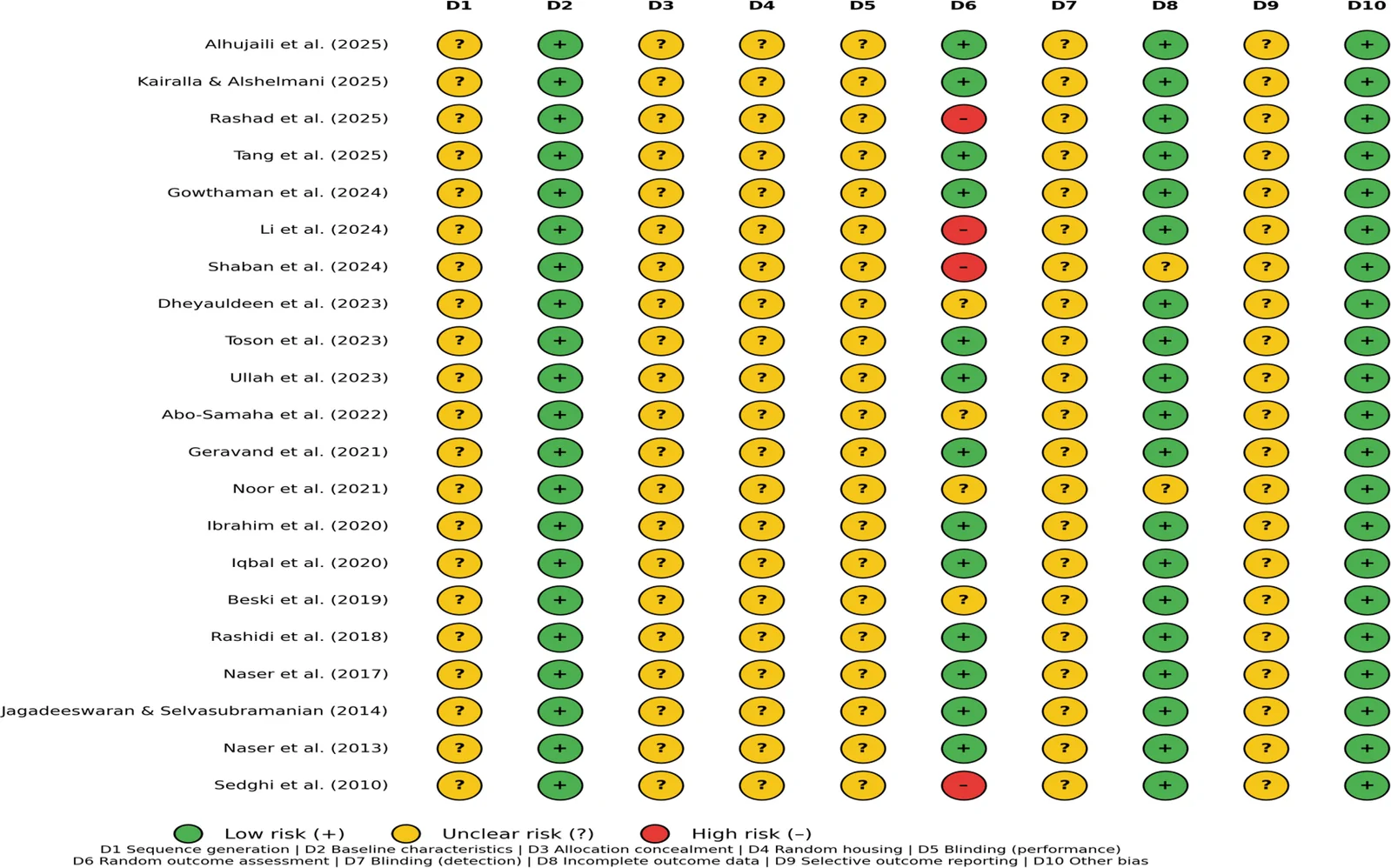
Per-study risk-of-bias assessment matrix.

**Fig. 4:**
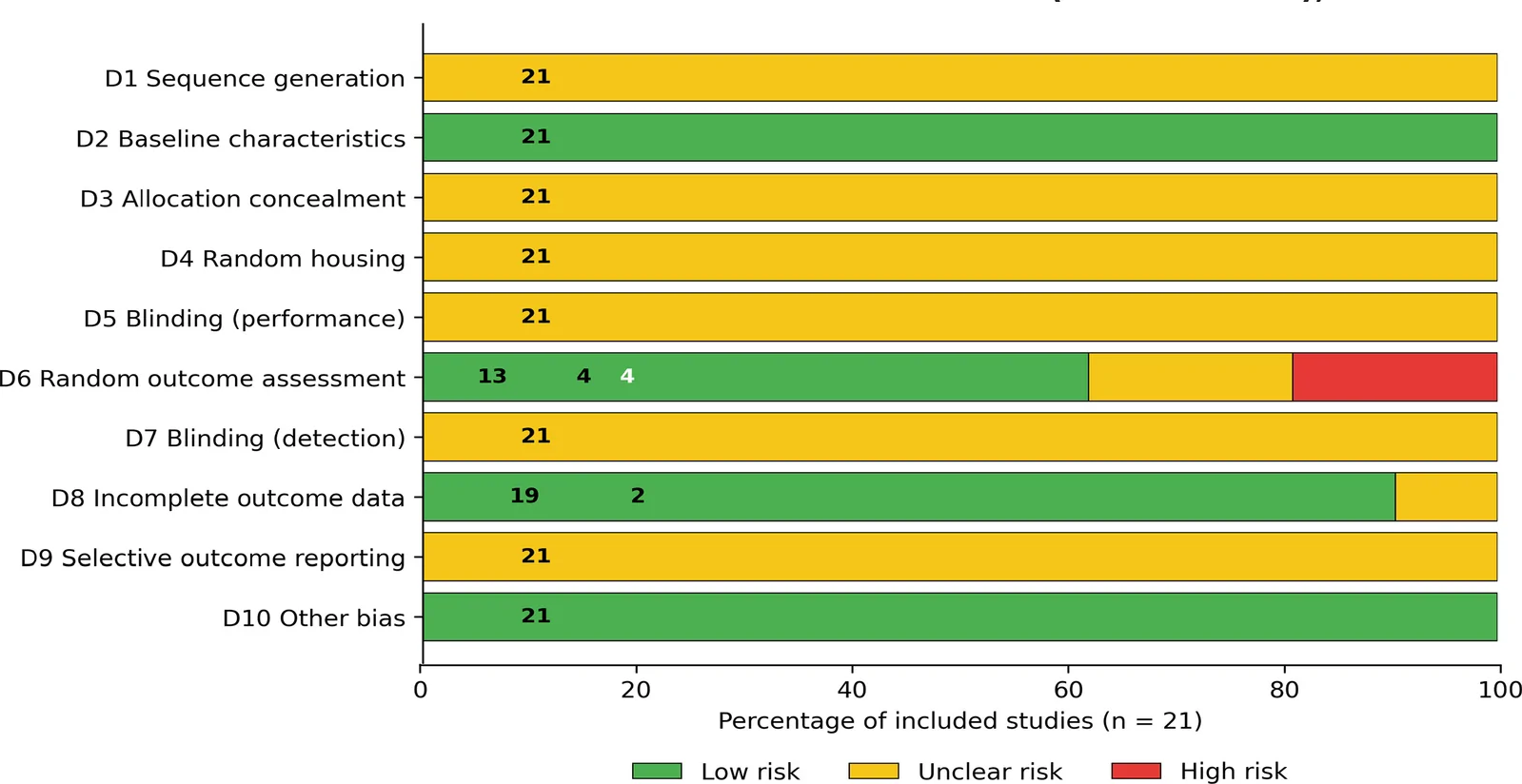
Risk-of-bias distribution across domains.

### Growth performance, carcass yield, visceral organs, intestinal morphology, and cecal bacterial count

Table 2 presents the pooled effects of dietary licorice on growth performance, carcass yield, visceral organs, intestinal morphology, and cecal bacterial count across all the included studies. Results indicated that dietary licorice significantly enhanced BWG, breast weight, thigh weight (Supplementary Fig. 1A), bursa of Fabricius, gizzard, VW, and VH/CD in broiler chickens compared to a control diet. While licorice significantly increased *Lactobacillus* spp. counts (SMD = 0.266; *P* = 0.003; 95% CI = 0.087 to 0.444; *I*^2^ = 27.93), its effect size also falls in the small range. In contrast, broiler chickens reared on diets containing licorice had significantly lower *Escherichia coli* (Supplementary Fig. 1B) than the control group. Nevertheless, dietary licorice did not vary FI, FCR, spleen, thymus (Supplementary Fig. 2A), liver, CD, or VH.

**Table 2:** Growth performance, carcass yield, visceral organs, intestinal morphology, and cecal bacterial count of broiler chickens supplemented with licorice.

Outcomes	*n*^s^	*n*^c^	SMD	95% CI		SE	*P*-value	Heterogeneity (%)			
				Lower	Upper			Q_M_	df	*P*-value	*I^2^*
*Growth performance*											
Feed intake (g)	19	53	0.034	−0.082	0.150	0.059	0.563	147.1	52	<0.001	65.53
BWG (g)	19	53	0.289	0.102	0.477	0.096	0.002	461.4	52	<0.001	86.58
FCR	21	56	−0.178	−0.367	0.011	0.096	0.064	534.6	55	<0.001	88.10
Carcass yield (g)											
Breast	8	22	0.757	0.550	0.964	0.106	<0.001	71.76	21	<0.001	69.24
Thigh	5	14	0.733	0.529	0.938	0.104	<0.001	24.76	13	0.025	44.54
Visceral organs (g)											
Spleen	8	21	0.627	−0.485	1.739	0.567	0.269	662.1	20	<0.001	98.91
Bursa of Fabricius	5	13	1.635	0.359	2.911	0.651	0.012	411.9	12	<0.001	98.09
Thymus	3	7	−0.992	−2.219	0.236	0.626	0.113	226.2	6	<0.001	97.77
Gizzard	8	22	0.861	0.432	1.291	0.219	<0.001	279.9	21	<0.001	92.20
Liver	14	39	0.233	−0.222	0.688	0.232	0.315	816.9	38	<0.001	96.57
*Intestinal morphology* (µm)											
Crypt depth	6	18	−0.477	−1.030	0.076	0.282	0.091	301.4	17	<0.001	93.81
Villus height	6	18	0.229	−0.425	0.883	0.334	0.493	204.6	17	<0.001	95.67
Villus width	4	11	1.055	0.227	1.884	0.423	0.013	186.0	10	<0.001	93.95
VH/CD	6	18	0.931	0.038	1.825	0.456	0.041	502.3	17	<0.001	97.17
*Bacterial counts* (Log cfu/g)											
*E. coli*	5	13	−1.737	−2.508	−0.966	0.393	<0.001	238.1	12	<0.001	96.15
*Lactobacillus* spp.	4	10	0.266	0.087	0.444	0.091	0.003	13.64	9	0.136	27.93

BWG = body weight gain; FCR = feed conversion ratio; VH/CD = villus height/crypt depth ratio; SMD = standardized mean difference; *n*^s^ = number of studies; *n*^c^ = number of comparisons; CI = confidence interval; Q_M_ = coefficient of moderators; SE = standard error; df = degree of freedom; *I²* = inconsistency index.

### Blood indices and oxidative status

Dietary licorice effects on blood indices and oxidative status are presented in Table 3. Meta-analysis results indicated that broiler chickens fed diets containing licorice had significantly increased GSH and catalase compared to a control group. Conversely, dietary licorice reduced ALT, AST (Supplementary Fig. 2B), cholesterol, glucose, and MDA. Broiler chickens fed diets with licorice showed no difference in the WBC, hemoglobin, RBC (Supplementary Fig. 3A), TP, albumin, globulin, and SOD (Supplementary Fig. 3B) compared to the control group.

**Table 3:** Blood indices and oxidative status of broiler chickens supplemented with licorice.

Outcomes	*n*^s^	*n*^c^	SMD	95% CI		SE	*P*-value	Heterogeneity (%)			
				Lower	Upper			Q_M_	df	*P*-value	*I*^2^
*Hematology*											
RBC (×10¹²/L)	5	14	−0.180	−2.919	2.558	1.397	0.897	409.0	13	<0.001	99.77
WBC (×10^9^/L)	5	13	0.143	−0.052	0.338	0.100	0.151	29.93	12	0.003	59.93
Hemoglobin (g/dL)	3	10	−0.018	−0.450	0.413	0.220	0.933	37.54	9	<0.001	80.66
*Liver enzymes*											
ALT (U/L)	7	21	−1.114	−1.853	−0.376	0.377	0.003	529.5	21	<0.001	97.49
AST (U/L)	7	21	−0.794	−1.530	−0.058	0.375	0.035	613.9	21	<0.001	97.50
*Serum biochemistry*											
Total protein (g/L)	10	27	−0.104	−0.535	0.327	0.220	0.635	492.9	26	<0.001	95.01
Albumin (g/L)	10	27	−0.228	−0.618	0.161	0.199	0.251	345.2	26	<0.001	94.06
Globulin (g/L)	10	27	−0.106	−0.759	0.546	0.333	0.749	900.4	26	<0.001	97.61
Cholesterol (mmol/L)	14	39	−0.920	−1.688	−0.153	0.392	0.019	236	38	<0.001	99.3
Glucose (mmol/L)	7	17	−2.013	−2.554	−1.473	0.276	<0.001	247.7	6	<0.001	94.40
*Oxidative status*											
GSH (mmol/L)	4	10	0.863	0.370	1.355	0.251	<0.001	100.1	9	<0.001	92.37
MDA (nmol/mL)	6	15	−3.695	−6.275	−1.114	1.317	0.005	1343	14	<0.001	99.62
SOD (U/mL)	5	13	−0.098	−2.054	1.858	0.998	0.922	731.1	12	<0.001	99.53
Catalase (U/mL)	3	10	1.207	0.676	1.738	0.271	<0.001	118.8	9	<0.001	92.13

ALT = alanine aminotransferase; AST = aspartate aminotransferase; GSH = glutathione; MDA = malondialdehyde; SOD = superoxide dismutase; SMD = standardized mean difference; *n*^s^ = number of studies; *n*^c^ = number of comparisons; CI = confidence interval; Q_M_ = coefficient of moderators; SE = standard error; df = degree of freedom; *I²* = inconsistency index

### Subgroup analyses

The subgroup analysis results observed heterogeneity in BWG between studies caused by variations in inclusion level, inclusion route, inclusion duration, form, sex, and strain (Table 4). Broiler chickens reared at 1–5 g/kg showed a significant increase in BWG. In contrast, broiler chickens that were fed diets containing 0–0.9 g/kg, 6–10 g/kg, and >10 g/kg of licorice had a comparable BWG to those reared on a control diet. Moreover, only the inclusion of licorice via feed increased BWG. Results indicate that broiler chickens reared on diets containing licorice for 1–35 days had increased BWG. However, birds reared on diets containing licorice for 1–28 days reduced BWG in comparison to those fed the control diet (Supplementary Fig. 4A). The results for the licorice form subgroup revealed that only the extract and powder enhanced BWG. Male and unsexed broilers fed a diet containing licorice showed a significantly increased BWG compared to those fed the control. The subgroup results revealed that only Ross strains reared on a diet supplemented with licorice had significantly higher BWG than the controls (Supplementary Fig. 4B).

**Table 4:** Effect of dietary licorice on the body weight gain of broiler chickens.

Moderators	Subgroup	*n*^s^	*n*^c^	SMD	95% CI		SE	*P*-value
					Lower	Upper		
Inclusion level (g/kg)	0–0.9	9	19	0.202	−0.070	0.474	0.14	0.145
	1–5	11	26	0.350	0.070	0.629	0.14	0.014
	6–10	4	4	0.103	−0.534	0.739	0.33	0.752
	>10	2	4	0.566	−0.575	1.707	0.58	0.331
Inclusion route	Drinking water	8	22	−0.063	−0.282	0.156	0.11	0.571
	Feed	11	31	0.539	0.293	0.785	0.13	<0.001
Inclusion duration (days)	1–28	1	2	−0.313	−0.602	−0.024	0.15	0.034
	1–35	7	22	0.577	0.286	0.867	0.15	<0.001
	1–42	9	24	0.011	−0.200	0.222	0.11	0.917
	>42	2	5	0.699	−0.128	1.527	0.42	0.098
Form	Extract	14	39	0.351	0.121	0.581	0.12	0.003
	Oil	2	5	−0.199	−0.779	0.380	0.30	0.500
	Powder	3	9	0.290	0.059	0.520	0.12	0.014
Sex	Male	2	5	1.244	0.570	1.918	0.34	<0.001
	Unsexed	17	48	0.177	0.008	0.347	0.09	0.041
Strain	Cobb	8	24	0.113	−0.098	0.325	0.11	0.293
	Kadaknath	1	4	0.317	−0.207	0.840	0.27	0.236
	Liangfenghua	1	1	2.048	1.714	2.382	0.17	NA
	Ross	7	19	0.489	0.174	0.804	0.16	0.002
	Unknown	2	5	−0.135	−0.755	0.484	0.32	0.668

*n*^s^ = number of studies; *n*^c^ = number of comparisons; SMD = standardized mean difference; CI = confidence interval; SE = standard error; *P*-value = probability value; NA = not available

Table 5 revealed subgroup results of the impact of the studied moderators on serum cholesterol in broiler chickens fed diets containing licorice. In comparison with the control, inclusion levels of 0–0.9, 1–5, and 6–10 g/kg decreased serum cholesterol in broiler chickens, whereas the >10 g/kg subgroup showed a significant increase in serum cholesterol. Likewise, only the inclusion of licorice via drinking water reduced serum cholesterol. Broiler chickens fed diets containing licorice for 1–28 days, 1–42 days, and >42 days had reduced serum cholesterol (Supplementary Fig. 5A). Only the inclusion of licorice, in the form of powder and oil, as well as supplementation for males, resulted in a reduction of serum cholesterol. The subgroup results revealed that only Cobb strains had comparable serum cholesterol (Supplementary Fig. 5B).

**Table 5:** Effect of dietary licorice on the serum cholesterol of broiler chickens.

Moderators	Subgroup	*n*^s^	*n*^c^	SMD	95% CI		SE	*P*-value
					Lower	Upper		
Inclusion level (g/kg)	0–0.9	7	12	−1.745	−2.691	−0.800	0.48	<0.001
	1–5	8	21	−1.298	−2.097	−0.499	0.41	0.001
	6–10	3	3	−0.961	−1.687	−0.235	0.37	0.009
	>10	1	3	5.352	3.845	6.859	0.77	<0.001
Inclusion route	Drinking water	4	10	−3.465	−4.323	−2.607	0.44	<0.001
	Feed	10	29	−0.048	−0.802	0.706	0.39	0.901
Inclusion duration (days)	1–28	1	2	−3.813	−7.608	−0.017	1.94	0.049
	1–35	4	12	−0.158	−2.261	1.945	1.07	0.883
	1–42	7	18	−0.781	−1.461	−0.102	0.35	0.024
	>42	2	7	−1.689	−2.683	−0.696	0.51	<0.001
Form	Extract	9	25	−1.076	−2.278	0.126	0.61	0.079
	Oil	2	5	−0.884	−1.389	−0.380	0.26	<0.001
	Powder	3	9	−0.476	−0.833	−0.119	0.18	0.009
Sex	Male	1	3	−2.941	−3.810	−2.073	0.44	<0.001
	Unsexed	13	36	−0.750	−1.556	0.056	0.41	0.068
Strain	Cobb	7	21	−0.877	−2.273	0.519	0.71	0.218
	Kadaknath	1	4	−0.728	−1.334	−0.122	0.31	0.018
	Ross	5	12	−1.011	−1.745	−0.278	0.37	0.007
	Unknown	1	2	−1.048	−1.517	−0.580	0.24	<0.001

*n*^s^ = number of studies; *n*^c^ = number of comparisons; SMD = standardized mean difference; CI = confidence interval; SE = standard error; *P*-value = probability value

### Meta-regression and publication bias

Table 6 shows a meta-regression analysis of the influence of moderators on BWG and serum cholesterol in broiler chickens fed diets containing licorice. The results of this meta-regression revealed that inclusion duration, inclusion route, sex, and strain had effects on BWG. However, only the inclusion level and the inclusion route had a moderating effect on serum cholesterol levels. Visual inspection of the funnel plot for BWG (Fig. 5) revealed a scattered distribution that was asymmetric around the pooled estimate. Egger’s regression test did not detect significant funnel plot asymmetry for BWG (*P* = 0.222) (Supplementary Fig. 6). Trim-and-fill analysis nonetheless imputed 7 missing studies and provided an adjusted 0.453 (95% CI = 0.234 to 0.671). Table 7 indicates that the Nfs for BWG were 792, which exceeded the robustness threshold of 105. The funnel plot for serum cholesterol (Fig. 6) revealed a closely clustered and asymmetric distribution. Egger’s regression detected significant funnel plot asymmetry (*P* = 0.042). However, trim-and-fill analysis estimated no missing studies and provided no adjusted mean effect size. The Nfs for serum cholesterol was 7562, exceeding the robustness threshold of 80 by 94.53-fold.

**Table 6:** Meta-regression of the associations between body weight gain and serum cholesterol and moderators.

Outcomes	Moderators	SMD	Q_M_	df	*P*-value	*I^2^* (%)	R^2^ (%)
Body weight gain (g)	Inclusion level (g/kg)	0.205	1.093	3	0.779	87.18	0.00
	Inclusion route	0.538	11.34	1	<0.001	83.85	19.13
	Inclusion duration (days)	0.011	13.36	3	0.004	83.60	19.43
	Form	0.350	2.861	2	0.239	86.18	2.21
	Sex	0.179	14.46	1	<0.001	82.71	25.18
	Strain	0.126	15.35	4	0.004	82.53	24.16
Cholesterol (mmol/L)	Inclusion level (g/kg)	−1.746	40.27	3	<0.001	98.68	50.23
	Inclusion route	−0.054	23.66	1	<0.001	98.91	38.90
	Inclusion duration (days)	−0.785	4.778	3	0.189	99.30	4.20
	Form	−1.085	0.384	2	0.825	99.35	0.00
	Sex	−0.751	2.330	1	0.127	99.30	3.52
	Strain	−0.888	0.045	3	0.998	99.36	0.00

SMD = standardized mean difference; Q_M_ = coefficient of moderators; df = degree of freedom; *I²* = inconsistency index; R² = proportion of heterogeneity explained by covariates.

**Fig. 5:**
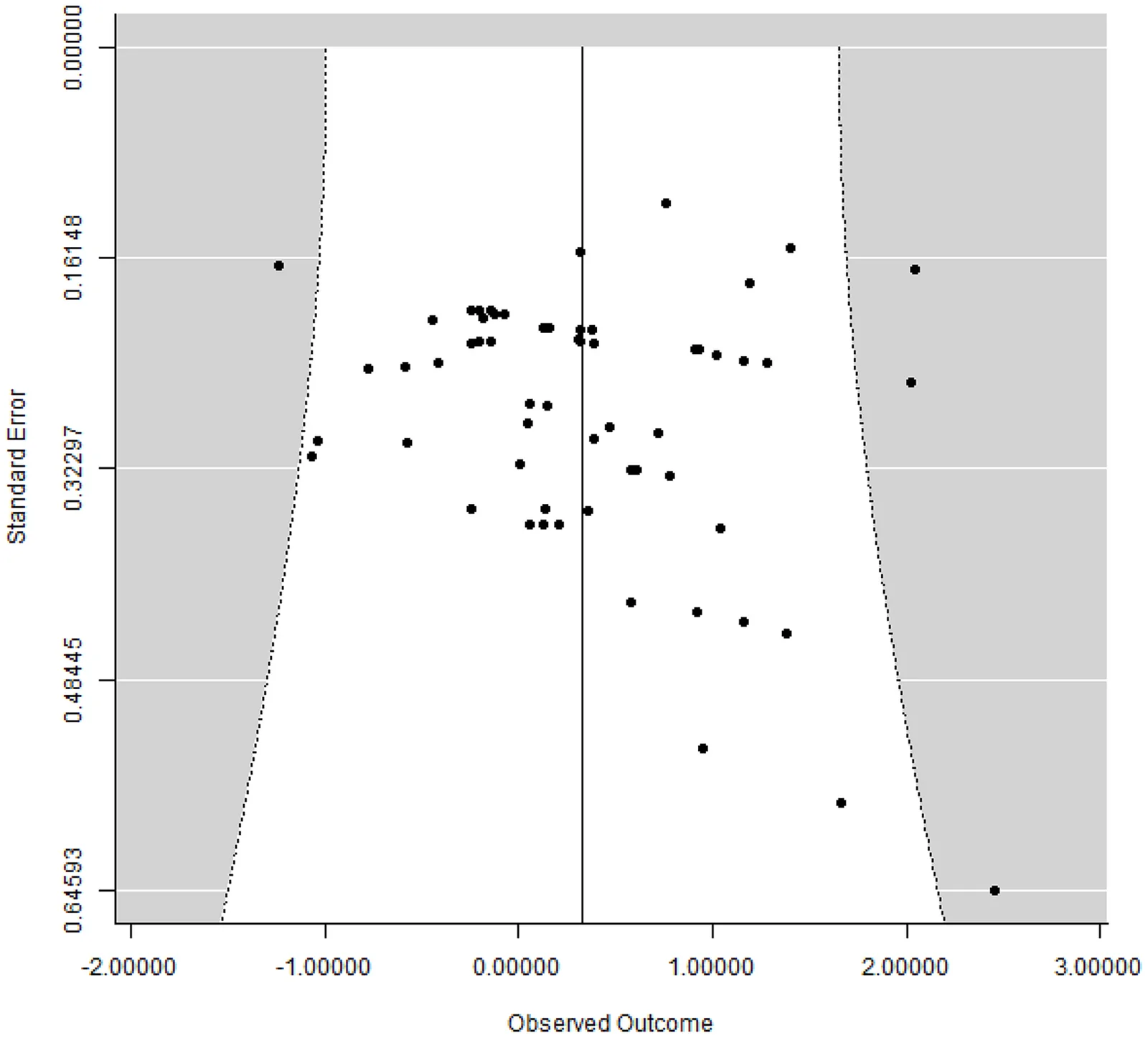
Body weight gain funnel plot.

**Table 7:** Analysis of publication bias.

Outcomes	SMD	*P*-value	*N*_fs_ number	NS	*N*_fs_>(5N_study_+10)
Body weight gain (g)	0.269	<0.001	792	19	105
Cholesterol (mmol/L)	−0.872	<0.001	7562	14	80

SMD = standardized mean difference; *N_fs_* = fail-safe number; NS = number of contributing studies.

**Fig. 6:**
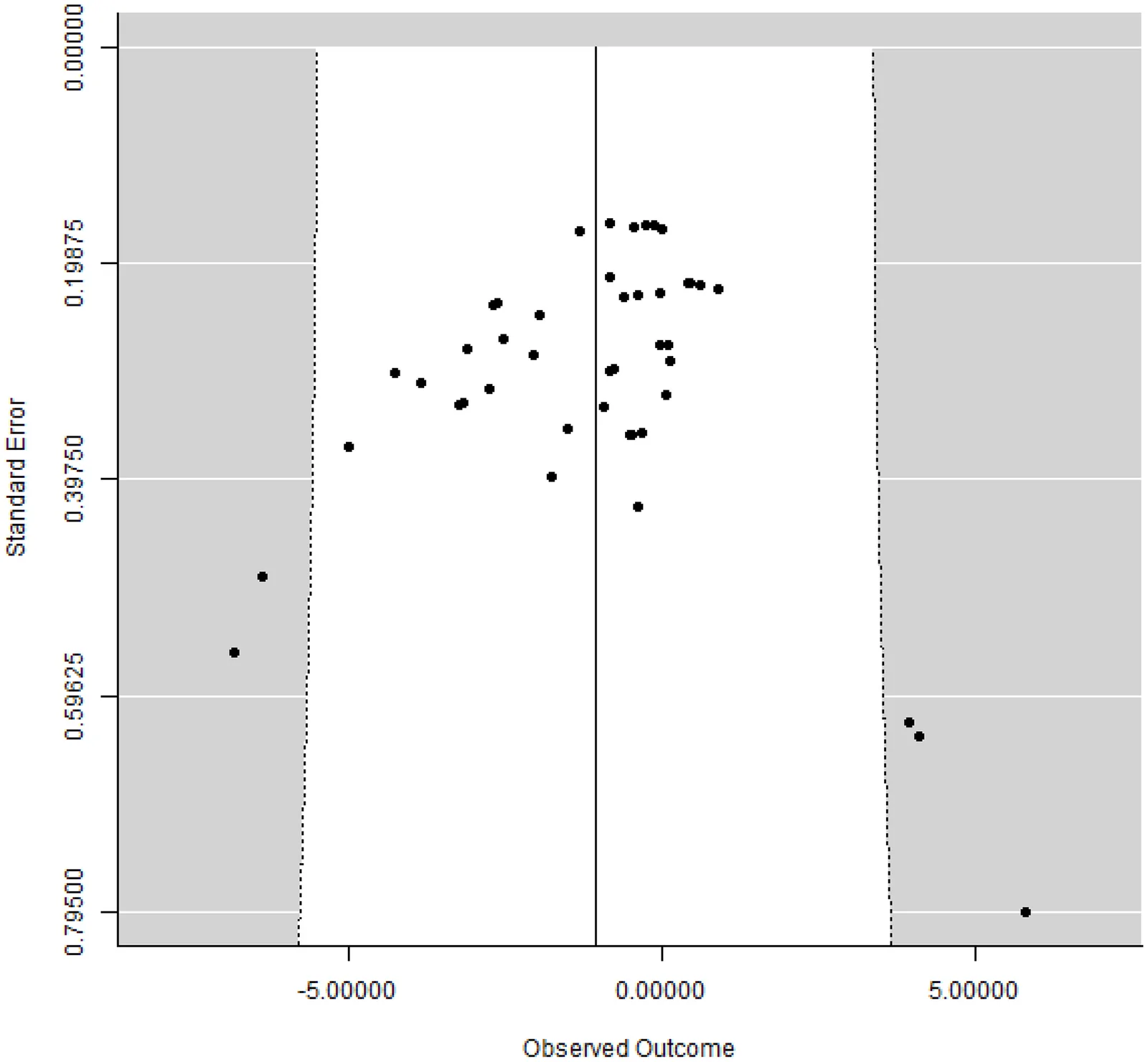
Serum cholesterol funnel plot.

## Discussion

Six of the ten domains were unclear across all 21 studies, indicating that the evidentiary base for this study is characterized by a pervasive reporting gap. This finding likely implies a convention in applied broiler nutrition trials. Generally, authors stated that the allocation was “random,” but they did not describe the randomization mechanism, allocation concealment, blinding, or the randomized housing procedures in full detail (Percie du Sert et al., 2020). Random outcome assessment showed that four studies selected "average-weight" animals for carcass and organ measurements rather than selecting at random. This practice can reduce the variations in these endpoints and skew the average values toward the group means (Festing, 2014). Furthermore, the lack of registered protocols across the 21 studies means the risk of selective outcome reporting cannot be excluded for any individual study (Higgins et al., 2019; Page et al., 2021). However, we found no evidence in the reported data indicating that any outcomes were measured but not reported.

### Growth performance, carcass yield, and visceral organs

In this study, dietary licorice did not influence FI and FCR but improved BWG. This could suggest that the bioactive compounds of licorice exert their growth-promoting effects through enhanced protein accretion (Shaban et al., 2024). Nevertheless, conventional benchmarks (Cohen, 1988) indicate that the magnitude of the FCR effect was small. This could suggest that licorice’s impact on feed efficiency, even if significant, is unlikely to be of translational importance to producers. Our findings are inconsistent with the study of Rashad et al. (2025), who noted no variation in the FI and BWG of unsexed Cobb 500 chickens after 35 days of supplementing with 3 g/kg licorice extract. The observed improvement in BWG in this study likely reflects enhanced intestinal barrier integrity. Strengthened tight junctions reduce paracellular permeability, limiting the translocation of luminal antigens and pathogenic bacteria, reducing the immunological burden on the bird, and redirecting metabolic energy toward tissue deposition. This is corroborated by Ibrahim et al. (2020), who reported that dietary 2 g/kg licorice extract increased BWG and feed efficiency by upregulating occludin, glucagon-like peptide, and junctional adhesion molecules in male Ross broilers. The strengthening of tight junctions reduces paracellular permeability, thereby limiting the translocation of luminal antigens and pathogenic bacteria, reducing the immunological burden on the bird and redirecting metabolic energy toward tissue deposition. Dietary licorice enhanced breast and thigh yields, which is consistent with the BWG improvement in this study, though between-study heterogeneity is high. This suggests the contributing studies reported conflicting estimates of the breast and thigh yields in response to licorice supplementation, likely reflecting differences in experimental conditions. Furthermore, the noted large confidence interval precludes any conclusions regarding the magnitude of the estimates for breast and thigh yields. This suggests more primary studies are needed to consolidate the evidence base for breast and thigh yields.

The bursa of Fabricius weight increased, while spleen and thymus weights did not vary in this study. This dissociation could suggest that licorice selectively potentiates the humoral immune system, given that the bursa is the primary site of B-lymphocyte maturation in birds (Liu et al., 2016). However, the high between-study heterogeneity implies that the pooled null effects for spleen and thymus weights should be interpreted cautiously. This selective bursal response is consistent with Sun et al. (2019), who reported that dietary dipotassium glycyrrhizinate, a water-soluble salt of glycyrrhizic acid derived from licorice root, enhanced the spleen, thymus, bursa of Fabricius, CD4⁺/CD8⁺ ratio, interferon-γ, and interferon-β levels in 24-day-old Hy-Line brown chicks. This supports the hypothesis that licorice promotes lymphoid organ development via enhanced cytokine-mediated immune activation. Likewise, dietary licorice extracts increased serum lysosomal activity in Cobb 500 broilers, probably suggesting an effect that boosts the immune system (Abo-Samaha et al., 2022). Lysosomes are essential to innate immunity mediated by macrophages (Hayama et al., 2018), further linking the bursa enlargement to a broader immune enhancement. While bursal enlargement is conventionally interpreted as evidence of enhanced B-cell immune competence (Ali et al., 2026), it is often a pathological precursor to severe immunosuppression (Singh et al., 2015). The enlarged gizzard, an organ of mechanical feed digestion, could suggest that licorice bioactive compounds support gizzard musculature activity and secretory function (Lentle et al., 2013), thereby improving BWG and breast and thigh yield.

### Intestinal morphology and cecal bacterial count

The VH/CD ratio is widely regarded as an indicator of intestinal absorptive capacity and mucosal maturity, as it integrates the surface area with the rate of crypt cell turnover (Belote et al., 2023). This study noted an enhancement in the VW and VH/CD ratio, while the VH and CD remained unchanged in response to the dietary licorice. This is likely implying that the ratio of mature absorptive surface to regenerative crypt activity was favorably shifted, which is consistent with the enhancements in BWG and carcass yield. Our study is inconsistent with the meta-analysis of Hernández-García et al. (2025), who reported improvements in VH, CD, and VH/CD in broilers fed diets containing curcumin. Chen et al. (2025) did report that 1 g/kg of licorice extract enhanced VW and the VH/CD ratio in male Liangfeng broilers exposed to low doses of deoxynivalenol and zearalenone. Indeed, dietary licorice reduced *Escherichia coli* counts and increased *Lactobacillus* spp. counts in this study. This result likely implies that dietary licorice exerts antibacterial effects through competitive exclusion dynamics, which could be attributed to the bioactive compounds of licorice (Babich et al., 2022). When coliform populations are suppressed, niche space and fermentable substrate availability increase for commensal lactic acid bacteria, whose proliferation further acidifies the intestinal milieu and perpetuates a hostile environment for intestinal pathogens. Moreover, the low and non-significant between-study heterogeneity for *Lactobacillus* spp. counts indicate that the increase is consistent across contributing studies. While the increase in *Lactobacillus* spp. was consistent across studies (low *I*²), its effect size was small. Consequently, this shift represents a subtle modulation rather than a complete change in the microbial balance of the cecum. We plausibly link this small but consistent increase to the enhanced intestinal morphology observed. A gut environment dominated by *Lactobacillus* spp. produces short-chain fatty acids, which serve as a trophic factor that provides the primary energy source for intestinal epithelial cells and modulate villus development (Liao et al., 2020).

### Blood indices and oxidative status

None of the measured hematological indices varied from the control group, despite very high *I*^2^ for RBC and hemoglobin. This null result likely indicates that, at the inclusion levels across the pooled trials, licorice does not compromise erythropoiesis or causes systemic toxicity. However, given the substantial *I*^2^ and the limited hematological indices evaluated, this finding should not be taken as evidence that licorice is free of systemic toxicity. Toxicological studies are warranted before firm safety conclusions are drawn. Our findings agree with the report of Kairalla & Alshelmani (2025), who noted no variation in the WBC, RBC, and hemoglobin of Cobb chickens fed diets containing 7.5 g/kg licorice for 42 days.

In the context of liver function, the reduced ALT and AST in this analysis could imply hepatocellular protection, which agrees with Abo-Samaha et al. (2022), who observed reduced ALT and AST with licorice extract supplementation in broiler chickens. The absence of a change in liver weight likely indicates that the hepatoprotective effect of licorice mitigated cellular damage and leakage (Kaur et al., 2026). Dietary licorice supplementation reduced serum cholesterol and glucose concentrations, while total protein, albumin, and globulin levels remained unaffected. This lipid-lowering effect could be attributed to its bioactive components, which inhibit hepatic HMG-CoA reductase activity and modulate lipid metabolism (Mahdavi et al., 2020). Furthermore, the concurrent drop in serum glucose suggests enhanced insulin sensitivity (Dheyauldeen et al., 2023), which likely facilitated the direct shunting of nutrients into skeletal muscle tissue, thereby supporting the observed improvements in BWG and breast and thigh yields. The reduction in serum glucose could be attributable to the hypoglycemic constituents of licorice, especially licochalcone A, which exhibits potent insulin-sensitizing properties (Luo et al., 2021). This concurrent reduction in cholesterol and glucose reflects a metabolic system that favors lean tissue accretion over lipid deposition and carbohydrate circulation, directly linking these serum biochemistry shifts to the improved BWG and carcass yields. Additionally, the absence of effects on total protein, albumin, and globulin may indicate that licorice does not impair hepatic synthetic function at the investigated inclusion levels (Giangrandi et al., 2024). This key safety inference is entirely coherent with the preserved relative liver weights and the qualitative hepatoprotection demonstrated by the suppressed ALT activity.

Dietary licorice increased GSH and catalase levels while reducing MDA. Nonetheless, SOD was not modulated. The reduction in MDA, a terminal product of polyunsaturated fatty acid peroxidation (Mohideen et al., 2023), likely indicates that licorice attenuated lipid peroxidation in broiler tissues. The concurrent elevation in GSH, an intracellular thiol-based antioxidant (Noguchi et al., 2023), and catalase, a hydrogen peroxide-scavenging enzyme (Moriwaki et al., 2021), establishes that licorice activated both non-enzymatic and enzymatic antioxidant pathways. These findings likely imply that dietary licorice reduces the oxidative modification of cell membranes across enterocytes, erythrocytes, and immune cells. This provides a clear explanation for the simultaneously observed improvements in intestinal absorptive morphology (increased VH/CD ratio and VW) and hepatoprotection (suppressed ALT and AST).

### Subgroup analysis

The subgroup analyses provided important insights into the sources of the substantial heterogeneity detected for BWG and cholesterol in the pooled effect analysis. For BWG, only inclusion levels of 1–5 g/kg enhanced BWG. Our results are likely to indicate a non-linear response, especially since the level of inclusion did not moderate BWG in the meta-regression. This finding likely reflects the inability of a linear moderator model to capture a non-monotonic inclusion effect. Thus, it reinforces the need for primary studies using an evenly graded range of inclusion levels to characterize this relationship more precisely. The bitter taste from its saponin and phenol content (Schmid et al., 2021) may explain reduced efficacy at the higher inclusion levels tested. This finding is consistent with the null effect for inclusion via drinking water, as liquid medium rushes through the digestive tract much faster than a solid-feed matrix (Abo-Samaha et al., 2022). For inclusion duration, 1–35 days improved BWG, which corresponds to the period of most rapid post-hatch intestinal maturation and microbiota establishment in broilers (Jha et al., 2019), when licorice could modulate a developing epithelium. By 1–42 days and >42 days, the gut and microbiota may have reached a stable equilibrium. Therefore, any early morphological advantage conferred by licorice may have been “caught up” by the control birds' own maturation, diluting the pooled effect at those inclusion durations. The reduced BWG at 1–28 days was based on one study and most likely reflects an early-life adaptation lag, such as a transient reduction in intake as chicks acclimate to the licorice taste (Schmid et al., 2021). The extract and powder forms, but not the oil form, improved BWG. This disparity likely reflects differences in bioactive concentration and bioavailability, given that the essential oil form lacks water-soluble glycyrrhizin and bioactive polysaccharide components (Geravand et al., 2021). Additionally, the enhanced BWG response observed in male birds reinforces an interaction between sex-specific anabolic physiology (Suliman et al., 2026) and the enhanced BWG effect of licorice.

The inclusion subgroup analysis for serum cholesterol indicates that the cholesterol-lowering efficacy of licorice triterpenoids is effective at levels of 0–0.9 g/kg, 1–5 g/kg, and 6–10 g/kg. Conversely, the reversal to hypercholesterolemia observed at >10 g/kg could be ascribed to excessive glycyrrhizin inhibiting 11β-HSD2, inducing a pseudoaldosterone-like state in which sustained glucocorticoid activation stimulates hepatic lipogenesis (Yoshino et al., 2021). Likewise, including licorice in drinking water reduced serum cholesterol, likely suggesting that drinking water sustained the metabolic hydrolysis of licorice’s bioactive compounds needed for hepatic regulation. Nonetheless, the limited number of contributing studies (*n* = 1) for the inclusion of licorice at >10 g/kg calls for further research. Notably, cholesterol reduction was significant for inclusion durations of 1–28 days, 1–42 days, and >42 days, but not for 1–35 days. This result could imply that a minimum cumulative exposure threshold is required to suppress hepatic cholesterol biosynthesis. A finding with direct practical implications for optimizing inclusion levels in commercial poultry operations. Oil and powder forms reduced cholesterol, while extract results did not differ, likely due to high variability in study formulations. The higher reductions noted for oil form could be due to its retaining lipophilic terpenoids and phytosterols that inhibit cholesterol absorption (Li et al., 2022), which are often lost in aqueous or ethanolic extraction processes (Yoon et al., 2025). The noted reduction in serum cholesterol for males alone is likely due to underpowered data (*n =* 1) and should be interpreted cautiously rather than as a robust sex-hormone-mediated lipid effect. However, Cobb strains alone showed no cholesterol reductions, suggesting potential strain-specific variations in hepatic lipid metabolism (Iqbal et al., 2023) or a limitation in subgroup statistical power.

### Meta-regression

The meta-regression analysis identified inclusion duration, sex, and strain as predictors of BWG variation. Surprisingly, the inclusion level was not a moderator of BWG, despite the observed moderate inclusion level’s significance in the subgroup analysis. The apparent discrepancy could suggest that a linear regression model does not adequately describe the non-linear dose–response relationship captured by the subgroup analysis. This emphasizes the necessity of complementary analytical strategies in meta-analytic inference. Conversely, for serum cholesterol, both the inclusion level and the route of inclusion emerged as predictors, with the inclusion level accounting for more between-study variance. This finding could suggest that the hypocholesterolemia response to licorice was driven by the inclusion level and route. Therefore, it is essential to provide a reliable quantitative framework that guides practical licorice supplementation recommendations for commercial feed formulation.

### Publication bias

The publication bias analysis via fail-safe number indicates that the pooled estimates for BWG and serum cholesterol are highly resistant to reversal by unpublished null results. This supports confidence in the reliability and generalizability of the results. Together, the meta-regression and publication bias analyses validate BWG and cholesterol as the primary reliable endpoints of licorice supplementation in broiler chickens and support their use as benchmark outcomes in future experimental and meta-analytic investigations. The observed funnel asymmetry for BWG with non-significant Egger regression and 7 "missing" studies imputed by trim-and-fill argues against publication bias. This pattern rather suggests the pooled effect for BWG was underestimated. On the other hand, the noted asymmetry for cholesterol, with significant Egger regression and no missing study by trim-and-fill, likely reflects between-study heterogeneity. Despite the *N*_fs_ values in this study suggesting that the pooled estimates are unlikely to be nullified by unpublished null findings, the *N*_fs_ has some recognized limitations. For instance, it does not account for effect size heterogeneity, assumes unpublished studies average a null effect, and can overstate robustness in the presence of small-study effects that inflate rather than obscure the pooled estimate (Rosenberg, 2005). Consequently, the BWG and cholesterol estimates should be interpreted with some caution regarding their true effect size. For the remaining outcomes, publication bias could not be formally assessed given the smaller number of contributing studies, which limits the reliability of inference based on funnel plots (Higgins et al., 2019; Sterne et al., 2011). This constrains how confidently these pooled estimates can be generalized and emphasizes the necessity of additional primary studies before firm practical recommendations are drawn.

Several limitations should be considered when interpreting the findings of our study. First, studies that met the inclusion criteria were from Africa and Asia only. Thus, the generalizability of our findings to broiler production systems outside these regions is therefore uncertain. Study quality was also uneven, with several subgroup strata, such as >10 g/kg licorice inclusion for BWG and male flocks for cholesterol, being represented by a single study. This limitation reduces the precision and statistical power of those specific estimates, necessitating cautious interpretation.

Secondly, because primary studies rarely reported standardized concentrations of bioactive constituents, dose normalization across preparations was impossible. Therefore, pooled dose–response relationships should be interpreted as indicative rather than precise. Differences in experimental conditions further compounded this variability, including inclusion level, route, duration, sex, and strain, each of which emerged as a moderator in our meta-regression analyses. Further indicating that pooled effects partly reflect the composition of the evidence base rather than a uniform biological response.

Thirdly, outcome measurement was not standardized across studies. For instance, assay methods for oxidative and biochemical markers varied, and study durations were grouped into non-uniform categories rather than analyzed as a continuous variable. This could obscure finer temporal dynamics of the licorice response. Consistent with this, several outcomes retained substantial between-study heterogeneity (*I*² > 50%) even after subgroup stratification. This could suggest that unmeasured moderators such as basal diet composition, housing and environmental conditions, and extraction solvent may also contribute to variability in effect sizes.

## Conclusion

This study provides quantitative evidence that dietary licorice exerts favorable effects on body weight gain, carcass yield, visceral organ development, gut health, and oxidative status in broiler chickens. Nevertheless, response magnitude for body weight gain was moderated by route, duration, sex, and strain. These findings support licorice as a viable phytogenic feed additive for antibiotic-free broiler production, although further research is needed to optimize inclusion strategies and validate benefits across diverse production systems.

## Ethics approval

The Animal Research Ethics Committee at the College of Agriculture and Environmental Sciences, University of South Africa, granted ethical approval (Ref: 2026/CAES_AREC/12898) for this meta-analysis.

## Consent for publication

Not applicable.

## Data statement

The datasets generated and analyzed during the current study are available in the Figshare repository: https://doi.org/10.6084/m9.figshare.33005750.

## Funding

Open access funding provided by the University of South Africa. The funders were not involved in the study’s design, data collection and analysis, publication decision, or manuscript writing.

## CRediT authorship contribution statement

**Chidozie F. Egbu:** Writing – review & editing, Writing – original draft, Visualization, Validation, Software, Resources, Methodology, Investigation, Formal analysis, Data curation, Conceptualization. **Christiaan J. Smit:** Writing – review & editing, Visualization, Validation, Supervision, Methodology, Investigation, Data curation, Conceptualization.

## Declaration of competing interest

The authors declare that they have no known competing financial interests or personal relationships that could have appeared to influence the work reported in this paper.
